# The effect of starting point placement technique on thoracic transverse process strength: an ex vivo biomechanical study

**DOI:** 10.1186/1748-7161-5-14

**Published:** 2010-07-13

**Authors:** Barrett S Brown, Terence E McIff, Rudolph C Glattes, Douglas C Burton, Marc A Asher

**Affiliations:** 1 Fondren Orthopedic Group, 7401 S Main St., Houston, TX 77030, USA; 2 Dept of Orthopedic Surgery, University of Kansas Medical Center, 3901 Rainbow Blvd, Kansas City, KS 66160, USA; 3 Elite Sports Medicine and Orthopaedic Center, 2021 Church St., Ste 200, Nashville, TN 37203, USA

## Abstract

**Background:**

The use of thoracic pedicle screws in spinal deformity, trauma, and tumor reconstruction is becoming more common. Unsuccessful screw placement may require salvage techniques utilizing transverse process hooks. The effect of different starting point placement techniques on the strength of the transverse process has not previously been reported. The purpose of this paper is to determine the biomechanical properties of the thoracic transverse process following various pedicle screw starting point placement techniques.

**Methods:**

Forty-seven fresh-frozen human cadaveric thoracic vertebrae from T2 to T9 were disarticulated and matched by bone mineral density (BMD) and transverse process (TP) cross-sectional area. Specimens were randomized to one of four groups: A, control, and three others based on thoracic pedicle screw placement technique; B, straightforward; C, funnel; and D, in-out-in. Initial cortical bone removal for pedicle screw placement was made using a burr at the location on the transverse process or transverse process-laminar junction as published in the original description of each technique. The transverse process was tested measuring load-to-failure simulating a hook in compression mode. Analysis of covariance and Pearson correlation coefficients were used to examine the data.

**Results:**

Technique was a significant predictor of load-to-failure (*P *= 0.0007). The least squares mean (LS mean) load-to-failure of group A (control) was 377 N, group B (straightforward) 355 N, group C (funnel) 229 N, and group D (in-out-in) 301 N. Significant differences were noted between groups A and C, A and D, B and C, and C and D. BMD (0.925 g/cm^2 ^[range, 0.624-1.301 g/cm^2^]) was also a significant predictor of load-to-failure, for all specimens grouped together (*P *< 0.0001) and for each technique (*P <*0.05). Level and side tested were not found to significantly correlate with load-to-failure.

**Conclusions:**

The residual coronal plane compressive strength of the thoracic transverse process is dependent upon the screw starting point placement technique. The funnel technique significantly weakens transverse processes as compared to the straightforward technique, which does not significantly weaken the transverse process. It is also dependent upon bone mineral density, and low failure loads even in some control specimens suggest limited usefulness of the transverse process for axial compression loading in the osteoporotic thoracic spine.

## Background

Hook anchors gained widespread acceptance with the development of Harrington instrumentation, and transverse process hooks are important components of most major implant systems. Their usage requires transverse processes sufficiently strong to bear the often substantial loads required to realign and stabilize the spine.

Pedicle screws allow delivery of large corrective loads to all three columns of the spine. This has led to their wider acceptance in thoracic spine deformity correction and stabilization [1]. However, the rate of misplaced thoracic screws ranges from 0% to 44% [2-10]. Some misplaced screws may be able to be salvaged. Others may require an alternate fixation method, such as a transverse process hook loaded in compression [11].

**Figure 1:**
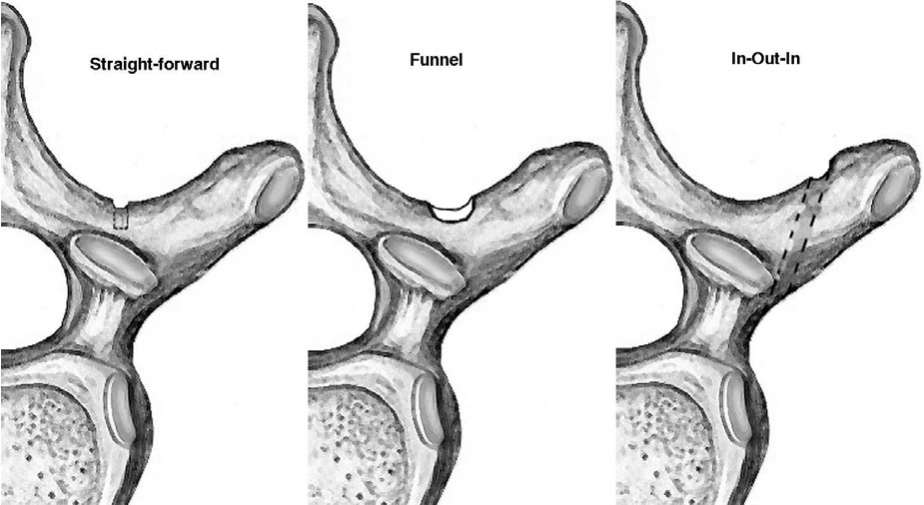
**The three techniques to start thoracic pedicle screw placement evaluated in this study: straight-forward, funnel, and in-out-in**.

There are three different techniques commonly used for thoracic pedicle screw placement. They can be distinguished by their different starting point location and cortical preparation. The straight-forward or freehand technique popularized by Suk [1] and Lenke [2,12] uses a starting point (Figure 1) in the region of the transverse process-laminar facet junction, typically just lateral to the vertical line bisecting the superior facet. The second major technique, known as the funnel technique and described by Gaines [3,13,14], utilizes a starting point (Figure 1) within the transverse process proper, creating a 6- to 10-mm defect in the posterior cortex. The third method, known as the in-out-in technique [15], is an extrapedicular placement technique. This utilizes a far lateral starting point (Figure 1) on the posterior cortex of the transverse process, perforates the ventral cortex of the transverse process, enters the lateral aspect of the pedicle, and passes into the vertebral body. This technique generally requires an extreme medially angled trajectory to achieve purchase in the vertebral body. Due to the differences in starting points and screw angulation, combining techniques on the same side of the spine can create difficulties for making rod connections and is therefore not generally recommended.

The purpose of this study is to evaluate the suitability of the transverse process as a fixation point following the use of the three starting points and preparation techniques described. Our hypothesis is that the biomechanical properties of the transverse process, in particular the coronal plane or compression bending strength, will be adversely affected when using certain pedicle screw starting point and preparation techniques.

## Methods

### Specimen Preparation

Forty-seven (47) fresh-frozen cadaveric thoracic vertebrae from 9 donor spines (T2-T9) were harvested and disarticulated. Not all thoracic levels from each donor spine were used due to previous fractures, abnormalities, or damage during specimen collection. The average age of donors was 72.1 years (range, 51-89 years), and the average age of vertebral body specimens was 71.8 years. Transverse process cranial-caudal and medial-lateral dimensions were measured at the midpoint of the transverse process using a digital caliper (± 0.01-mm resolution). Each thoracic vertebral segment subsequently underwent evaluation with DEXA scanning to assess bone mineral density (BMD) while immersed in rice, as suggested by the equipment manufacturer (GE Lunar Prodigy, GE Medical Systems, Madison, WI).

The vertebrae were divided into four study groups: (A) control, (B) straight-forward, (C) funnel, and (D) in-out-in. In the control group no entry site was made, and the transverse processes were left intact. In the treatment groups, entry site placement and starting point techniques were done as described in the literature: the straight-forward (Figure 1) as described by Kim et al. [2], the funnel technique (Figure 1) as described by Yingsakmonkol et al. [3], and the in-out-in technique (Figure 1) as described by Belmont et al. [15]. Each transverse process was assigned to one of the four study groups. Two of the groups were assigned to each vertebra, one group to the left TP and another to the right TP. All possible combinations based on inverting and mirroring the treatment assignments ensured no side- or size-related bias.

For all techniques, posterior cortical defects corresponding to the assigned treatment were created by a single surgeon in each transverse process with the same 4-mm round, fluted burr. A gearshift awl was used to cannulate the starting point just to the region of the pedicle entry site. This was easy to judge as the specimens were mounted in a vise that allowed excellent visualization of the outer walls of the pedicle and spinal canal. Neither were the pedicle tubes entered nor breeches made in the pedicle walls. No additional probing or tapping was performed. No screw was ever placed.

In both the straight-forward and in-out-in techniques, the posterior cortical hole diameter was limited to the 4-mm burr diameter. For the funnel technique, the posterior cortical defect was enlarged using the 4- mm burr to between a 9-mm and 10-mm diameter. For the in-out-in technique, a gearshift awl was additionally used to perforate the ventral cortex of the transverse process in a trajectory that would allow entry of the screw through the lateral wall of the pedicle into the vertebral body, but no such vertebral body breech was made. The initial placement of the hole was more lateral than that of the straight-forward and funnel techniques as required by the need to exit the transverse process on the ventral side.

### Testing/Measurement

**Figure 2:**
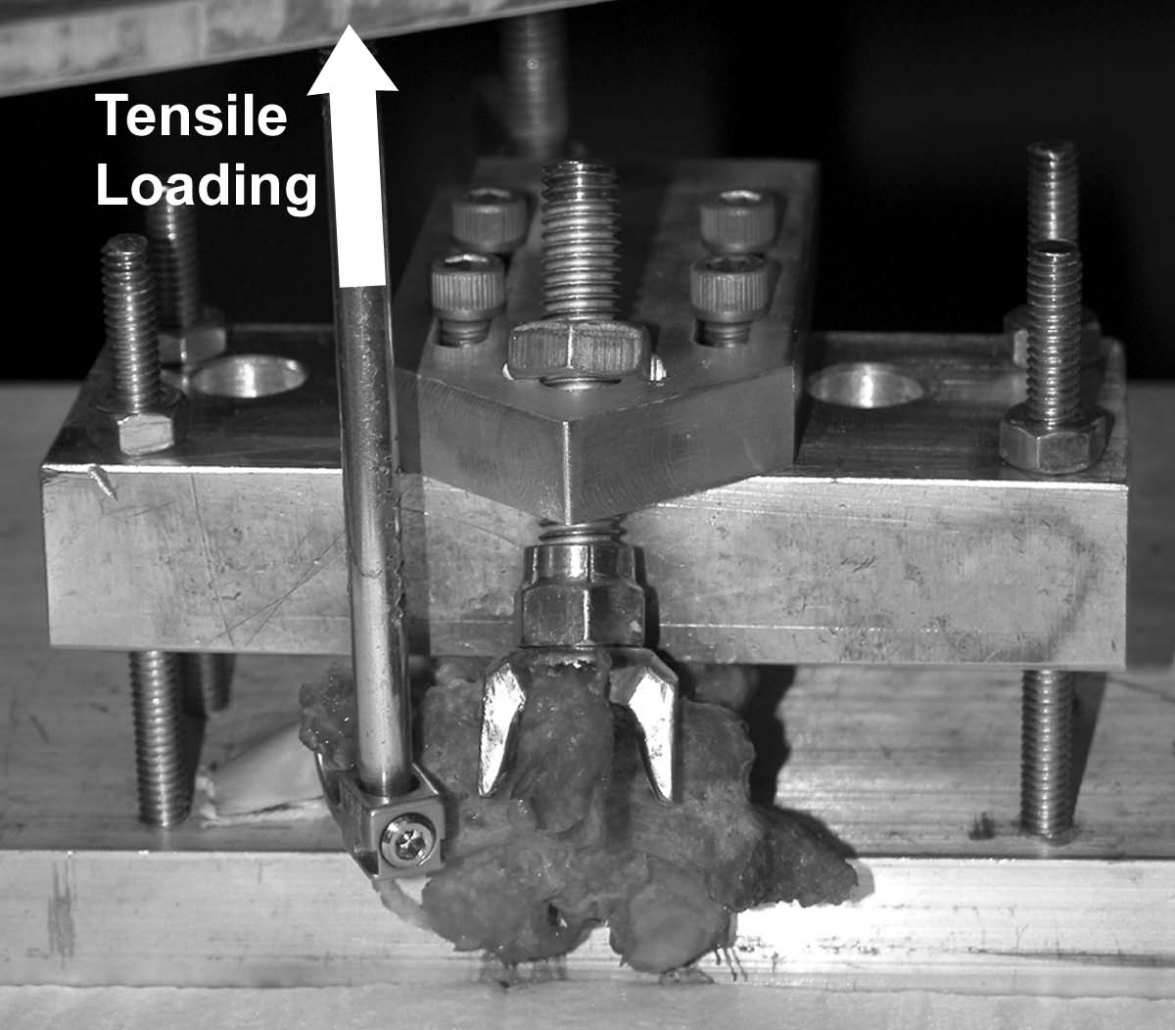
**Custom fixture holding each vertebral body rigidly while an upward (caudally directed) force was applied to the end of the TP using a standard hook**. Vertebrae were held inverted with the spinous process supported.

The specimens were secured to the load cell of a servohydraulic testing machine (MTS Mini-Bionix 858, Eden Prairie, MN) using a custom fixture specifically designed for this project (Figure 2). Each vertebral body was securely held by the fixture in an inverted orientation (caudal up). In addition, the posterior elements (superior and inferior lamina) were stabilized by an adjustable support arm, allowing isolation of forces to the transverse process. Secure fixation at the level of the vertebral body as well as stabilization of the posterior elements at the lamina demonstrated adequate stabilization of the vertebral body in preliminary testing.

Each transverse process was loaded to failure by an upward (caudally) directed load using a standard TP hook attached to the actuator of the testing machine. Clinically, this is the equivalent of compressive loading. For each side and specimen tested, the transverse process hook was placed 6 mm from the TP-facet junction. Data, including the load-to-failure and mode of failure, were collected. Load-to-failure data were analyzed using an analysis of covariance (ANCOVA) with technique as a categorical predictor and both BMD and TP area as continuous predictors (Statistica, StatSoft Inc., Tulsa, OK). Vertebral level was stratified to allow inclusion in the model. Pearson correlation coefficients were calculated as needed. Post-hoc comparisons were made using Fisher LSD. The statistical significance was set at 0.05 for both correlation and comparison.

## Results

Three vertebrae were eliminated from testing due to technical problems or structural insult to the pedicles during preparation. Forty-four vertebrae were ultimately tested, giving a total of 88 TP tests, using the TP on both sides of each vertebra. The number of transverse processes tested at each vertebral level was 8 (T2), 8 (T3), 8 (T4), 12 (T5), 12 (T6), 14 (T7), 12 (T8), and 14 (T9). Failure primarily occurred by bending and fracture of the TP at the site of the defect. Failure always occurred by fracture of the TP at the site of the defect in specimens using the funnel and in-out-in techniques (44 of 44). Fracture through the lamina occurred in the case of some intact TP only. In tests of the straight-forward technique, 9 failures occurred lateral to the defect, 2 through the pedicle, and 12 through the defect. Failure through the defect occurred more frequently in specimens having lower BMD than in those having higher BMD.

Based on analysis of variance both technique (*P *= 0.0007) and BMD (*P *< 0.0001) were found to be significant predictors of load-to- failure. TP area (*P *= 0.057) was found to be a near significant predictor. Level was not found to be a significant predictor and was removed from the final model (*P *= 0.727).

**Figure 3:**
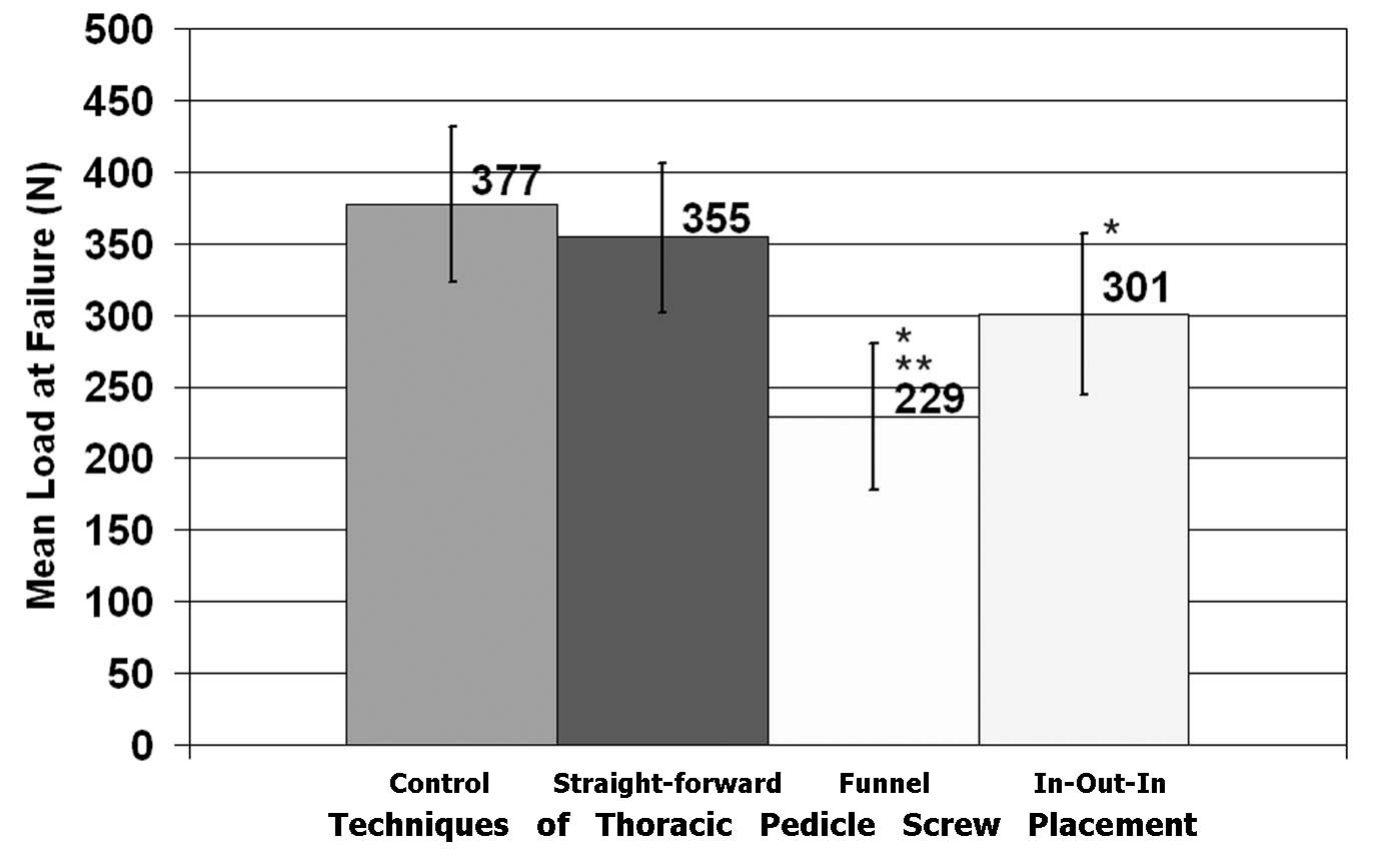
**Mean load-to-failure for each of the four study groups**. Error bars represent 95% confidence intervals of the estimated means. *Significantly different from intact control, *P *< 0.05. **Significantly different from straight-forward and significantly different from in-out-in, *P *< 0.05.

The least squares mean (LS mean) load-to-failure of group A (control) was 377.2 N (95% CI: 322.2-432.2, n = 21), group B (straightforward) 354.5 N (CI: 302.0-406.9, n = 23), group C (funnel) 229.0 N (CI: 177.7-280.3, n = 24), and group D (in-out-in) 301.2 N (CI: 245.1-357.4, n = 20). The LS means were calculated for the covariates (BMD and TP area) at their means. The LS means for each technique and their 95% confidence intervals are illustrated in Figure 3. The lowest single load-to-failure values were recorded for the funnel technique (61 N, BMD = 0.708 g/cm^2 ^and 71 N, BMD = 0.761 g/cm^2^). The lowest load-to-failure found for intact TP was 118 N (BMD = 0.761 g/cm^2^).

Post-hoc comparisons revealed significant differences in the mean load-to-failure between the control (A) and funnel (C) groups (*P *< 0.0001), the control (A) and in-out-in (D) groups (*P *= 0.026), the straight-forward (B) and funnel (C) groups (*P *= 0.001), and the funnel (C) and in-out-in (D) groups (*P *= 0.036). The straight-forward and control means were not found to be significantly different (*P *= 0.199), and the straight-forward and in-out-in means were similarly not found to be significantly different (*P *= 0.301).

**Figure 4:**
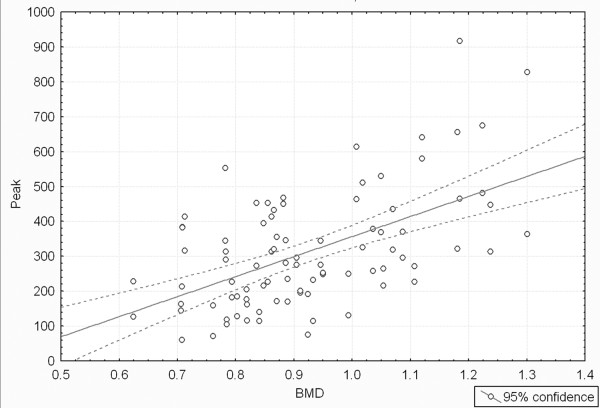
**Load-to-failure versus BMD showing regression line and 95% confidence lines (r = 0.55, correlation is significant, *P *< 0.0001)**.

The mean BMD for all specimens was 0.925 g/cm^2 ^(range, 0.624- 1.301 g/cm^2^). BMD significantly correlated with load-to-failure of all specimens grouped together (r = 0.55, *P *< 0.0001, Figure 4). When the load-to-failure data were grouped by pedicle screw technique, the correlation was significant (*P *< 0.05) for each technique. The Pearson correlation coefficients for BMD and load-to-failure by group were 0.66 (A), 0.52 (B), 0.65 (C), and 0.59 (D), which were all significant.

Mean TP cross-sectional area for all specimens was 118.1 mm^2 ^(range, 88.6-193.1 mm^2^). When grouped by technique, TP cross-sectional area was significantly correlated (r = 0.55, *P *< 0.05) with load-to-failure only for group A (control). Neither level nor side (right or left pedicle) significantly correlated with load-to-failure (*P *> 0.05).

## Discussion

In this study, we measured load-to-failure of transverse processes in the thoracic spine after simulation of pedicle screw placement using one of three described techniques. Our hypothesis was that the technique used to prepare the thoracic pedicle screw placement site could adversely affect transverse process strength, a fixation site that might be needed if pedicle screw placement was unsatisfactory. Our data supported this hypothesis, most notably when comparing the straight-forward and funnel techniques, residual transverse process strength being significantly stronger following straight-forward preparation than funnel (355 N *vs*. 229 N). Only after straight-forward preparation was the transverse process strength not significantly different than the control, 355 N *vs*. 377 N. Though the number of specimens tested was not large enough to demonstrate significant differences between these two groups, the difference in mean load-to failure (22 N) between the straight-forward technique and the control is small and not likely to be clinically relevant.

Successful clinical use of the straight-forward technique has been reported by many authors. Kim et al. [2] reported placing over 3,200 screws in a "step-wise, consistent and compulsive manner" without any neurological, vascular, or visceral complications at up to 10 years' follow-up. They felt this technique was a safe, accurate, and reliable method of placing thoracic pedicle screws.

This study identifies an upper bound on the failure strength of transverse processes following preparation for pedicle screw placement. In fact, neither were pedicle screws inserted nor the holes tapped, a step that would have contributed further to disruption of the load-resisting structure. The smallest diameter of burr (4 mm) was used in the straight-forward technique, and no additional cortical bone was removed as is often done to allow for proper seating of the screw head. However, the same small 4-mm burr was also used to create the hole for the in-out-in technique. Though no screw was placed, each technique did include creation of a deeper hole into the cancellous bone by use of the gearshift awl. The creation of a larger diameter entry hole, tapping of the hole, insertion of a screw, or countersinking of the hole would have further weakened the cortical bone and led to lower recorded transverse process strength.

Observation of the fracture site in each specimen revealed that a large number that were compromised using the straight-forward technique fractured lateral to the burr hole (9 of 23). Those using the funnel or in-out-in technique always fractured through the defect (44 of 44). This is evidence that the intact transverse process strength often dominates when the defect is small and placed more medially, but when the cortical defect extends more laterally, the defect strength dominates, and process strength is substantially reduced.

BMD was found to have a very substantial effect on the transverse process strength. This was the case even though the BMD values represented whole body BMD rather than just local transverse process BMD. This finding is similar to that of other investigators who have documented the effect of BMD on thoracic pedicle screw pullout strength [16] and the failure strength of other spine fixation devices and constructs [17]. The effect of TP cross-sectional area was found to be significant but less substantial than that of vertebral body BMD. TP cross-sectional area was only significantly correlated with load-to-failure for the control specimens. We have concluded that the defects, once created, overshadowed any remaining effect of TP cross-sectional area on TP strength.

We believe this study provides the only data available in the literature on the strength of transverse processes, either intact or compromised by defects, under loads replicating those applied by TP hooks. This information is useful for understanding the load limitations of current and potential devices. The lowest load-to-failure recorded during our series was 61 N, which occurred with the funnel technique and in conjunction with one of the lowest BMD values. Although the average failure load for our intact control specimens was 377 N, one intact transverse process from one of our lower BMD vertebral bodies failed at 118 N. Such very low values are worth remembering when contemplating the use of TP hooks in osteoporotic thoracic spines.

Perhaps the most obvious limitation of this study is that pedicle screws were not actually placed. Our rationale for the experimental methodology used was to produce reproducible preparation sites with accuracy. An extension of this experiment would be to actually place the pedicle screws, a step that could only be predicted to further weaken the transverse process. Another criticism is the advanced age of the cadaveric specimen donors. Though the average age was high, the average BMD was nearly normal (0.925 g/cm^2^). Also, we did not explore the use of alternative fixation techniques, including the use of wiring, cabling, and supralaminar hooks for the upper foundation. Since most surgeons experienced with deformity, trauma, and tumor reconstruction have some background experience utilizing transverse process hooks, this model was chosen. Future studies may examine the use of alternative fixation when the pedicle is not accessible and the transverse process has been compromised.

The values calculated occurred in a very controlled and isolated testing environment. In the in vivo environment, the applied loads may well be distributed along the segmentally instrumented spine. However, the initial compressive force when setting the hook in surgery should mimic our experimental design. Additionally, we did not evaluate the effect of submaximal forces that may create microfractures of the transverse process and create potential problems over time before the arthrodesis has healed.

## Conclusion

The entry site preparation for thoracic vertebra for pedicle screw placement significantly (*P *= 0.0007) affects the residual coronal plane compression strength of the transverse process. Strength is least affected by the straight-forward technique and most affected by the funnel technique. Bone mineral density is also a significant (*P *< 0.0001) predictor of transverse process load-to-failure strength. These findings raise doubt about the suitability of some transverse processes as spine implant fixation sites.

## Competing interests

MAA owns 30% of Isola Implants Inc.

DCB is a consultant to DePuy Spine.

No other authors have competing interests.

## Authors' contributions

BB participated in the study design, carried out specimen preparation, participated in data acquisition, and contributed to manuscript drafting. TM participated in data acquisition, data analysis, interpretation of data, and manuscript drafting. CG participated in the design of the study, interpretation of data, and manuscript drafting. DB participated in the design of the study, interpretation of data, and manuscript drafting. MA participated in the design of the study and manuscript drafting. All authors read and approved the final manuscript.
